# Ruminative self-focus, negative life events, and negative affect

**DOI:** 10.1016/j.brat.2008.06.004

**Published:** 2008-09

**Authors:** Nicholas J. Moberly, Edward R. Watkins

**Affiliations:** Mood Disorders Centre, School of Psychology, University of Exeter, Exeter EX4 4QG, UK

**Keywords:** Experience-sampling, Affect, Rumination, Stressors, Depression

## Abstract

Ruminative thinking is believed to exacerbate the psychological distress that follows stressful life events. An experience-sampling study was conducted in which participants recorded negative life events, ruminative self-focus, and negative affect eight times daily over one week. Occasions when participants reported a negative event were marked by higher levels of negative affect. Additionally, negative events were prospectively associated with higher levels of negative affect at the next sampling occasion, and this relationship was partially mediated by momentary ruminative self-focus. Depressive symptoms were associated with more frequent negative events, but not with increased reactivity to negative events. Trait rumination was associated with reports of more severe negative events and increased reactivity to negative events. These results suggest that the extent to which a person engages in ruminative self-focus after everyday stressors is an important determinant of the degree of distress experienced after such events. Further, dispositional measures of rumination predict mood reactivity to everyday stressors in a non-clinical sample.

## Introduction

Dysfunctional mood regulation has been highlighted as a key factor in the onset and maintenance of psychological distress. Particular interest has centred on rumination, defined by Nolen-Hoeksema (1991, p. 569) as “repetitively focusing on the fact that one is depressed; on one's symptoms of depression; and on the causes, meanings, and consequences of depressive symptoms”. According to response styles theory (RST), a ruminative response style prolongs sad mood relative to engagement in pleasant, distracting activities (Nolen-Hoeksema, 1991, 2000). Considerable evidence suggests that dysphoric rumination exacerbates negative mood and negative cognition (see Lyubomirsky & Tkach, 2004; Watkins, 2008).

A related line of investigation addresses how rumination influences emotional reactions to stressful life events. Controlling initial levels of depression, a ruminative response style predicts future depressive symptoms in response to events such as the Lomo Prieta earthquake (Nolen-Hoeksema & Morrow, 1991) or the death of a loved one (Nolen-Hoeksema, Parker, & Larson, 1994). Similarly, Robinson and Alloy (2003) reported that rumination on negative inferences after stressful events (*stress-reactive rumination*) interacted with dysfunctional attitudes and negative attributional style to predict new depressive episodes among students.

Despite a large body of research suggesting that stressful life events are associated with short-term increases in negative affect (Marco & Suls, 1993; Suls, Green, & Hillis, 1998; Swendson, 1998), little is known about the role of rumination in this process. Thus, the first aim of this study was to address how negative affect fluctuates after everyday stressors, and whether levels of momentary ruminative self-focus mediate this relationship. The second aim was to investigate whether individuals who report high levels of trait rumination (high ruminators) show greater reactivity to negative life events than low ruminators.

Many studies examining the influence of rumination on reactivity to stressful events have used retrospective assessments that may not accurately reflect how individuals responded shortly after the stressful event (Stone et al., 1998). Daily diary designs represent an improvement in this regard (e.g., Wood, Saltzberg, Neale, Stone, & Rachmiel, 1990), but often require that reports of response styles are made several hours after their occurrence, increasing the probability of retrospective bias.

In experience-sampling methodology (ESM; Csikszentmihalyi & Larson, 1987) studies, the participant provides ‘online’ data about experience as prompted by an alarm signal. Retrospective bias is thus virtually eliminated, although ESM remains susceptible to other response biases that are inherent in self-report measures (Stone et al., 1998). Furthermore, ESM can track fluctuations in affect and ruminative self-focus over relatively short temporal intervals, enabling the researcher to examine contingencies of which the participant may be unaware.

Peeters, Nicolson, Berkhof, Delespaul, and deVries (2003) used ESM to investigate the effect of positive and negative life events on mood among depressed individuals and non-depressed controls. Although negative events were associated with increased negative affect in both groups, depressed participants were less reactive to negative events than non-depressed participants. Swendson (1998) similarly found that negative events were associated with negative affect among undergraduates, but this relationship was not moderated by depressive symptomatology.

Given that rumination is normally conceptualized as a response to negative mood, one might expect rumination to moderate and/or mediate the impact of distressing events on psychological distress. In an ESM study of adolescents, Silk, Steinberg, and Morris (2003) found that the use of involuntary engagement strategies (including rumination) after negative events was associated with greater sadness and anger at a subsequent occasion, and that higher levels of involuntary engagement over the week were associated with more depressive symptoms. Unfortunately, this study did not uniquely address ruminative thinking.

We conducted an ESM study in which adults reported their negative affect, ruminative self-focus, and negative events eight times daily for one week. On each occasion, we measured momentary negative affect as a composite of sadness, anxiety, and irritation ratings, on the basis that rumination is associated with each of these affects (Blagden & Craske, 1996; Nolen-Hoeksema, 2000; Rusting & Nolen-Hoeksema, 2000). Participants indicated on each occasion whether or not they had experienced a recent negative event, provided a brief description of this event, and indicated how emotionally distressing it was.

We assessed momentary ruminative self-focus using a two-item measure comprising the extent to which people were focused on (i) their feelings and (ii) their problems. The first item corresponds to the facet of Nolen-Hoeksema's (1991) definition involving focus on depressive feelings. The second item corresponds to the facet of Nolen-Hoeksema's (1991) definition implicating focus on causes and consequences of depression. Focus on problems matches discrepancy-based accounts suggesting that unresolved problems underlie rumination (Lyubomirsky, Tucker, Caldwell, & Berg, 1999; Martin & Tesser, 1996).

We hypothesised that negative affect would be associated with (i) negative events that were reported concurrently and (ii) prior negative events that were reported at the previous occasion. Second, we hypothesized that negative affect would be predicted by momentary ruminative self-focus at the previous occasion. Third, because emotionally negative events tend to induce ruminative self-focus (Wood, Saltzberg, & Goldsamt, 1990), we hypothesized that ruminative self-focus would partially mediate the association between prior negative events and negative affect. Fourth, informed by RST, we hypothesised that trait rumination would moderate the association between negative events and negative affect such that high trait ruminators would report more negative affect after negative events than low trait ruminators. However, on the basis of previous findings, we did not expect levels of depressive symptomatology to moderate the association between negative events and negative affect.

## Method

### Participants

Participants were recruited from the University of Exeter and the local area using e-mails and newspaper advertisements. We requested volunteers for a study on sad moods and depression, although we made it clear that participants did not have to be depressed to take part. Thus, as intended, we obtained a sample with a wide range of depressive symptomatology as assessed on the Beck Depression Inventory-II (range = 0–37, *M* = 15.4, SD = 9.2). One hundred and thirty-nine persons (100 women) initially consented to take part (range = 18–67 years, *M* = 26.8, SD = 13.3). Most (107) were university students, the remainder were community adults. Data from a subset of these participants examining the direct relationship between negative affect and ruminative self-focus were previously reported by Moberly and Watkins (2008). Participants were paid £10 ($20) for completing the study.

### Measures

#### Beck Depression Inventory-II (BDI-II)

The BDI-II assesses levels of depressive symptomatology with 21 items that are rated on a scale from 0 to 3, with higher scores reflecting more depressive symptoms (range = 0–63) (Beck, Steer, & Brown, 1996). Cronbach's alpha for our sample was 0.90.

#### Response Styles Questionnaire–Ruminative Responses Scale (RSQ)

The RSQ assesses the extent to which individuals respond to depressed mood by focusing on self, symptoms and on the causes and consequences of their mood (trait rumination), using 22 items rated on a 4-point frequency scale (Nolen-Hoeksema & Morrow, 1991). Cronbach's alpha for our sample was 0.91.

### Procedure

We used ESM to assess negative affect, ruminative self-focus and negative events eight times daily over seven days. Participants rated their moods and thinking styles when signalled by an alarm from a wrist-worn actiwatch (Cambridge Neurotechnology Ltd., Cambridge, UK). Each participant's day was divided into eight equal periods so that one alarm occurred at a random time within each period, and no two alarms occurred within 15 min. We sampled eight times daily to capture a range of psychological states across sufficient time points to enable sensitivity to changes in time and setting, without over-burdening the participant, as typical of similar experience-sampling studies (Marco & Suls, 1993). This resulted in a 12 hr daily sampling period with one alarm occurring within each of eight 90 min periods (e.g., 10.00–22.00). Times were individually randomised for each participant to suit their typical waking hours (actual range = 07.00–23.59).

At each alarm, a flashing letter on an LED display prompted participants to enter a rating for the moment before the alarm sounded, by pressing a button on the actiwatch to cycle through ratings from 1 to 7. After each rating was entered, the next letter was displayed and the participant made the next rating. The actiwatch only accepted entries within 20 s of each alarm, ensuring all data were entered promptly. Participants recorded their levels of sadness (S), anxiety (N), and irritation (I), and the extent to which they were focusing on their feelings (F) and focusing on their problems (P) on a 7-point scale from 1 (*not at all*) to 7 (*very much*). During the study, participants carried a card on their person to remind them of the meaning of these prompts.

Participants received separate booklets for each day of the study. Each booklet included eight experience-sampling forms, each of which corresponded to an actiwatch alarm. Spaces were provided for participants to record (a) time and date of form completion, and (b) elapsed time since the alarm. Printed below was the question: ‘*Since the last beep, have you experienced an event that made you feel negative emotions?*’, which participants answered by circling either ‘*Yes*’ or ‘*No*’ and writing down a brief description of the event if it occurred. Scales were provided on which participants could rate the extent to which they felt *sad*, *anxious*, and *irritable when the negative event occurred* from 1 (*not at all*) to 7 (*very much*). To examine persisting effects of negative events, we identified negative events as *prior* when the previous ESM report on the same day (time *t* − 1) mentioned a negative event.

At an initial briefing session, participants completed baseline measures of trait rumination and depressive symptoms. Next, participants were shown the actiwatch and experience-sampling forms, and participants practised responding to a hypothetical alarm. We emphasised that the actiwatch questions referred to the moment just before the alarm sounded. The participant then chose the beginning and end of the sampling period, and this information was used to configure the actiwatch. After the sampling week, participants returned the actiwatch and forms to the laboratory, before being paid and debriefed.

### Treatment of experience-sampling data

Data were excluded from twenty-two participants who withdrew from the study during the week of experience-sampling (*n* = 13, ESM was too time-consuming; *n* = 5, actiwatch malfunctioned; *n* = 1, illness; *n* = 1, family emergency; *n* = 1, experienced mood recording as upsetting; *n* = 1, ESM interfered with therapy). Data were also excluded for occasions when the participant failed to complete the watch and form ratings within 15 minutes. Timely completion of the experience-sampling forms was verified with reference to (i) the reported time of form completion and (ii) the reported time interval between the actiwatch signal and form completion. Following standard guidelines (Delespaul, 1995), 11 participants who responded to less than one-third of the alarms within 15 min were excluded from the analysis. These non-completers did not differ significantly from completers on BDI-II score, RSQ score, gender or age.

Data from 106 participants (78 women) were analysed (age range = 18–67 years, *M* = 25.7 years, SD = 12.7)^1^ The mean response rate to the actiwatch alarms was 82.6% (SD = 10.9%) and the mean completion rate for the experience-sampling forms was 63.4% (SD = 15.4%). The total number of occasions that were validly recorded and analysed was 3775.

We calculated a composite measure of momentary negative affect by standardizing each of the sad, anxious, and irritated ratings and summing the resulting *z*-scores (*α* = 0.70). We calculated a composite measure of momentary ruminative self-focus by standardizing the focus on feelings and focus on problems ratings and summing the resulting *z*-scores (*α* = 0.67).

### Multilevel modelling

In our data structure, occasions (Level 1) were nested within days (Level 2) and within persons (Level 3). We used hierarchical linear modelling to investigate the relationships within and between different levels without violating assumptions of independence (Snijders & Bosker, 1999), using MLwiN v.2.02 software to conduct our analyses (Rasbash, Steele, Browne, & Prosser, 2005).

Our main analysis examined the concurrent and prospective association between negative events (recorded at time *t* and prior time *t* − 1) and negative affect (recorded at time *t*). To do this, we constructed a multilevel model using a subset of the dataset (2459 occasions) for which two subsequent occasions were recorded. We did not include a lagged measure of negative affect (recorded at time *t* − 1) because it was highly likely to be correlated with the random parts of the multilevel model, thereby violating a key modelling assumption (Spencer, 2002).

In our model, the intercept was specified as randomly varying at both the day and person levels, reflecting the fact that observations tend to be more similar if they are (a) taken on the same day, and (b) taken from the same person. All occasion-level predictors were modelled with coefficients that were randomly varying at the person level, to allow the relationship between negative events, ruminative self-focus, and negative affect to vary between individuals.

Trait dispositional variables and momentary ratings of ruminative self-focus were entered as continuous explanatory variables centred on the grand mean. Linear and quadratic variables for time of day (measured in days and centred on the mean sampling time, 15:04) and linear variables for day of study (centred on day 4) were also included as covariates.

## Results

### Negative events

Participants recorded 652 negative events. Using pre-established criteria (Peeters et al., 2003), the first author categorised each event as external if it had been prompted by a past event or situation that occurred outside the person, or internal if it had not. Internal events included unprompted thoughts, ruminations, and worries. A final-year psychology undergraduate independently categorised all negative events, yielding 93% category agreement with the first coder, *κ* = 0.77. Remaining differences were resolved through discussion. This process identified 128 internal events (19.6%). Results were similar whether or not internal events were excluded, and so results including all events are reported.

Negative event frequency was significantly correlated with BDI-II score, *r*(106) = 0.24, *p* = 0.01, but not with RSQ score, *r*(106) = 0.12, *p* = 0.24. Mean negative event severity (calculated by standardizing and summing the event-specific ratings of sadness, anxiety and irritation) correlated positively with BDI-II score, *r*(93) = 0.32, *p* < 0.01, and RSQ score, *r*(93) = 0.42, *p* < 0.001. RSQ score was associated with negative event severity when controlling BDI-II score, *r*(90) = 0.29, *p* < 0.01, but BDI-II score was not associated with negative event severity when controlling RSQ score, *r*(90) = 0.08, ns.^2^ Negative event frequency and mean negative event severity were not significantly correlated, *r*(93) = −0.06, ns.

### Negative events and concurrent negative affect

We first modelled negative affect with linear and quadratic effects of time and linear effects of day to control for temporal variation in negative affect and reduce the autocorrelation between successive observations. There was a significant linear effect of time on negative affect, *B* = −0.787, SE = 0.368, *p* < 0.05, such that negative affect tended to decrease over the course of the day. No other fixed effects were significant. Inclusion of the time and day variables resulted in a significant improvement in model fit over the null model, change in log-likelihood *χ*^2^(8) = 51.41, *p* < 0.001.

Subsequently, to account for individual differences in mean levels of negative affect, we simultaneously added the person-level variables of depressive symptomatology and trait rumination. Both depressive symptoms (*B* = 0.485, SE = 0.128, *p* < 0.001) and trait rumination (*B* = 0.039, SE = 0.013, *p* < 0.01) were associated with mean levels of negative affect. The addition of these variables resulted in a significant improvement in model fit, *χ*^2^(2) = 48.55, *p* < 0.001.^3^

To test our first hypothesis that negative events would be positively associated with negative affect at the contemporaneous sampling occasion (time *t*), we entered a dichotomous variable indicating whether or not participants reported a negative event at time *t*. In the same step, we entered negative event frequency and its interaction with negative events, to test whether negative affect was associated with negative event frequency and whether individuals who reported more frequent negative events would be more or less reactive to such events. As hypothesized, negative events reported at time *t* were associated with higher levels of concurrent negative affect at time *t*, *B* = 2.202, SE = 0.469, *p* < 0.001. Frequency of negative event reports was unrelated to mean levels of negative affect, *B* = 0.952, SE = 0.674, ns. Negative events interacted significantly with negative event frequency to predict negative affect, *B* = −1.956, SE = 0.977, *p* < 0.05, indicating that individuals reporting more frequent negative events were less reactive to these events than individuals reporting less frequent negative events. Inclusion of these terms resulted in a significantly improved model fit, *χ*^2^(21) = 236.53, *p* < 0.001.

### Negative events and prospective negative affect

In the next step, we tested our hypotheses that negative events and ruminative self-focus at the *previous* sampling occasion (time *t* − 1) would each predict negative affect at the *subsequent occasion* (time *t*). We entered a dichotomous variable indicating whether or not a *prior* negative event had been reported at time *t* − 1 and a continuous variable representing *prior* momentary ruminative self-focus at time *t* − 1. The report of a prior negative event at time *t* − 1 was associated with higher levels of negative affect at time *t*, *B* = 0.447, SE = 0.111, *p* < 0.001. Higher levels of prior ruminative self-focus at time *t* − 1 were also associated with higher levels of negative affect at time *t*, *B* = 0.152, SE = 0.029, *p* < 0.001. The inclusion of prior negative events and prior ruminative self-focus significantly improved the model fit, *χ*^2^(13) = 100.83, *p* < 0.001.

We next tested the hypothesis that prior momentary ruminative self-focus mediated the prospective effect of prior negative events on negative affect (Baron & Kenny, 1986). First, we tested the path from the initial variable to the putative mediator, by constructing a model with prior ruminative self-focus as the criterion variable. This revealed that prior negative events predicted prior ruminative self-focus, *B* = 0.836, SE = 0.102, *p* < 0.001. Second, we tested the direct path from the initial variable to the criterion variable in the absence of the mediator. Prior negative events predicted negative affect when ruminative self-focus was not included in the model, *B* = 0.580, SE = 0.110, *p* < 0.001. Third, as shown earlier, the mediator predicted the outcome variable: prior ruminative self-focus was significantly associated with negative affect. Fourth, the relationship between the initial variable and the outcome variable was reduced when the mediator was included: the magnitude of the prior negative event coefficient reduced from 0.836 to 0.447 when prior ruminative self-focus was included (see previous analysis of the association between prior negative events at time *t* – 1 and levels of negative affect at time *t*). Conditions for partial mediation were met (Sobel test, *z* = 4.44, *p* < 0.001).

For multilevel models with lower-level path coefficients that vary randomly at a higher level, Kenny, Korchmaros, and Bolger (2003) warn that calculation of the indirect path must consider the covariance between the higher-level random effects. Using their procedure, in which OLS estimates for the indirect paths are calculated for each person, we estimated the covariance between the person-level random effects to be 0.03. Because this covariance and the path coefficients for the indirect path were positive, our prior calculation of the indirect effect was an underestimate: 26% (rather than 22%, according to the standard approach) of the total effect of prior negative events on negative affect was mediated by prior ruminative self-focus.^4^

**Fig. 1:**
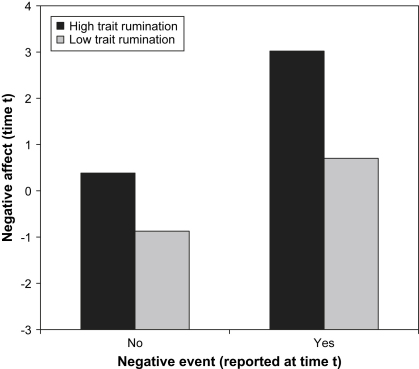
Relationship between reported occurrence of a negative event and negative affect (both reported at time *t*) for individuals scoring high (one SD above the mean) and low (one SD below the mean) on trait rumination (RSQ). Negative affect is a summed composite of the standardized ratings for individual items.

In a final step, we tested the hypothesis that trait rumination would moderate the impact of negative events on negative affect, but depressive symptomatology would not. To do this, we simultaneously included cross-level interactions between each of the person-level variables (BDI-II and RSQ) and (i) negative events (time *t*) and (ii) prior negative events (time *t* − 1). Coefficients for this final model are shown in Table 1. The interaction between trait rumination and negative events (time *t*) was associated with negative affect, but no other interaction was significant. Consistent with RST, high ruminators experienced greater negative affect after negative events than low ruminators (at time *t*; see Fig. 1). The interactions between depressive symptomatology and negative events were not significantly associated with negative affect. Inclusion of all interactions resulted in a significant improvement in model fit, *χ*^2^(4) = 14.01, *p* < 0.01.^5^

When internal negative events were excluded, the interaction between depressive sympatomatology and prior negative events became statistically significant, *B* = −0.228, SD = 0.115, *p* < 0.05, such that persons reporting more depressive symptomatology were less reactive to prior negative events. All other significant findings remained unchanged.

## Discussion

Our finding of an association between negative events and negative affect replicates the results of other diary and experience-sampling studies (Marco & Suls, 1993; Peeters et al., 2003; Swendson, 1998). Although we found that prior negative events predicted negative mood up to three hours later, evidence from other ESM studies has been inconsistent. Marco and Suls (1993) failed to find any effect on mood of prior negative events occurring on average 90 minutes previously, while Peeters et al. (2003) found that prior negative events were associated with negative affect for individuals in a major depressive episode but not for non-depressed individuals. By contrast, van Eck, Nicolson, and Berkhof (1998) found that prior stressful events were associated with increased negative affect in community adults. Differences in the operationalization of negative events may account for these divergent findings.

Our study demonstrates that momentary ruminative self-focus partially mediates the relationship between prior negative events and momentary negative affect. The absence of evidence for an interaction between prior negative events and ruminative self-focus testifies to the adverse consequences of ruminative thought in many circumstances, and not only after stressful events (see Moberly & Watkins, 2008). Our mediation analysis suggests that one reason why negative events are distressing in the short-term is because they induce ruminative thinking, which itself has depressogenic consequences (Lyubomirsky & Nolen-Hoeksema, 1993). To our knowledge, this study is the first to have used online measures with sufficient temporal resolution to identify this role of ruminative self-focus in emotional reactivity after negative events.

We found that trait ruminators experienced greater negative affect after negative events that were reported at the same occasion. This result is congruent with Robinson and Alloy's (2003) finding that the combination of elevated trait rumination and a stressful event is associated with increased distress. Trait rumination did not interact with prior negative events to predict subsequent negative affect, possibly because prior momentary ruminative self-focus was already included in the model. Interestingly, trait ruminators reported their negative events as more severe than other individuals did, even after controlling for depressive symptomatology. Though a ruminative tendency may make negative events seem worse, interpretation of this finding is complicated by the possibility that event severity judgments were influenced by the elevated levels of negative affect that high ruminators recorded when the negative event was reported.

Replicating Swendson's (1998) results, we found no evidence that dysphoric individuals were especially reactive to negative events. In fact, when internal events were excluded, dysphoric persons were *less* reactive to prior negative events than others. Peeters et al. (2003) found that clinically depressed participants were less reactive than non-depressed controls immediately after negative events, but were more reactive to negative events that occurred at the previous sampling occasion. Relatedly, laboratory studies have found that depressed individuals show reduced emotional reactivity to personally relevant negative material (Rottenberg, 2005).

Although negative event frequency was not uniquely associated with negative affect, participants with more depressive symptoms reported more frequent negative events, replicating diary studies with non-clinical samples (e.g., Grosscup & Lewinsohn, 1980). Interestingly, individuals reporting a high frequency of negative events were also less reactive to these events. This is unlikely to be due to more liberal event-reporting criteria, because frequency and mean event severity were not significantly correlated. Though requiring replication, this finding suggests that emotional response may become desensitized after repeated negative life events.

This study has several limitations. First, the sample consisted mainly of undergraduates, who may differ from other adults in the events they experience and their reaction to these events. Second, the demanding ESM protocol means that our volunteers may have been more conscientious and self-focused than the wider population (Scollon, Kim-Prieto, & Diener, 2003). Advertising the study as relevant to depression is likely to have resulted in volunteers with more depressive symptoms than randomly selected individuals. However, because it is debatable whether clinical depression differs dimensionally or categorically from non-clinical depression (Flett, Vredenburg, & Krames, 1997), it is unclear whether our findings would generalize to a clinically depressed sample.

Other concerns relate to the adequacy of our experience-sampling procedure. To minimise participant burden over repeated assessment, we used only three items to measure negative affect and two items to measure ruminative self-focus. Although the composites demonstrated acceptable internal consistency and validity (see Moberly & Watkins, 2008), future research would ideally use a more comprehensive set of items to capture these constructs, within the constraints of ESM. Excluding occasions when participants failed to respond within 15 minutes may have resulted in an under-representation of certain everyday situations (e.g., driving). Relatedly, we could not verify participants' reports of when the experience-sampling form was completed. ‘Backfilling’ is relatively common in diary and experience-sampling studies (Stone, Shiffman, Schwartz, Broderick, & Hufford, 2002). Future studies could use electronic personal data assistants to record the time of data entry more precisely.

Because participants recorded negative events after rating their negative affect, concurrent associations between these variables cannot establish that negative events *caused* negative affect. Nevertheless, our finding that *prior* negative events predicted subsequent negative affect does suggest that negative events have a prospective influence on negative affect. Finally, our multilevel analysis prevented us from controlling for prior levels of negative affect. Although we designed our composite measure of ruminative self-focus to be independent of negative affect, it is possible that the influence of prior ruminative self-focus was overestimated.

Our study provides further support for Nolen-Hoeksema's (1991) RST in relation to everyday stressful events. Momentary ruminative self-focus partially mediated the association between prior negative events and negative affect, while trait rumination moderated the association between negative life events and negative affect. By developing our understanding of mood regulation in an ecologically valid setting, we hope these results will contribute to the development of therapeutic approaches that improve psychological resilience.

## Figures and Tables

**Table 1:** Fixed effects estimates for negative affect

Predictor	Coefficient (SE)
Person-level variables	
BDI-II	0.431 (0.124)**
RSQ	0.031 (0.012)*
NEP	0.473 (0.640)

Momentary variables	
NE	2.111 (0.422)***
PNE	0.472 (0.111)***
PRSF	0.151 (0.028)***

Cross-level interactions	
NEP × NE	−1.802 (0.875)*
BDI-II × NE	−0.145 (0.139)
RSQ × NE	0.043 (0.013)**
BDI-II × PNE	−0.202 (0.114)
RSQ × PNE	0.010 (0.010)

*Note*. Analyses include 2459 occasions. Model includes linear and quadratic effects of time of day, and linear effect of day. Asterisks indicate that the coefficient differs significantly from 0. BDI-II = Beck Depression Inventory-II, RSQ = Ruminative Response Scale (total score), NEP = proportion of occasions on which a negative event was reported, NE = negative event (reported at time *t*), PNE = prior negative event (reported at time *t* − 1), PRSF = prior ruminative self-focus (reported at time *t* − 1). *p < 0.05. **p < 0.01. ***p < 0.001.

## Footnotes

1 The small number of males resulted in low statistical power to detect gender effects and so we did not include this variable in our analyses.

2 Neither the RSQ brooding (*α* = 0.72) nor the reflective pondering (*α* = 0.74) subscale scores (Treynor, Gonzalez, & Nolen-Hoeksema, 2003) were significantly associated with negative event frequency. Brooding was associated with negative event severity, *r*(93) = 0.36, *p* < 0.001, but reflection was not, *r*(93) = 0.12, ns. Brooding was significantly associated with negative event severity after controlling for BDI-II score, *r*(90) = 0.24, *p* < 0.02.

3 When the RSQ brooding and reflective pondering subscales were substituted for the total score, brooding was associated with higher mean levels of negative affect, *B* = 0.147, SE = 0.048, *p* < 0.01, as was depressive symptomatology, *B* = 0.538, SE = 0.117, *p* < 0.001. Reflective pondering was not significantly associated with mean levels of negative affect, *B* = −0.019, SE = 0.040, ns.

4 Although the dynamic, inter-correlated nature of our variables suggest that a mediating (rather than moderator) role for ruminative self-focus is most appropriate, we also tested the interaction between prior negative events and prior ruminative self-focus in a subsequent step, but the coefficient was not significant, *B* = −0.038, SE = 0.049, ns, and its inclusion did not significantly improve model fit, *χ*^2^(1) = 0.55, ns.

5 When RSQ brooding and reflective pondering subscale scores were entered instead of the RSQ total score, brooding interacted significantly with both negative events, *B* = 0.186, SE = 0.051, *p* < 0.001, and prior negative events, *B* = 0.082, SE = 0.042, *p* < 0.05, indicating that negative events were more strongly predictive of negative affect for brooders. The only other significant cross-level interaction was that prior negative events were less strongly associated with negative affect for individuals who reported greater depressive symptomatology, *B* = −0.240, SD = 0.108, *p* < 0.05.
